# Analysis of Colon Transcriptomes in a Porcine Model of Dextran Sodium Sulfate (DSS)-Induced Ulcerative Colitis

**DOI:** 10.3390/biology15141123

**Published:** 2026-07-10

**Authors:** Dan Hao, Xiao Wang, Guangqiang Shang, Aysevil Pektas, Stig Purup, Bo Thomsen

**Affiliations:** 1 Jinan Key Laboratory of Poultry Germplasm Resources Innovation and Healthy Breeding, Poultry Institute, Shandong Academy of Agricultural Sciences, Jinan 250100, China; 2 Department of Molecular Biology and Genetics, Aarhus University, 8000 Aarhus, Denmark; 3 Institute of Animal Science and Veterinary Medicine, Shandong Academy of Agricultural Sciences, Jinan 250100, China; 4 Department of Animal and Veterinary Sciences, Aarhus University, 8830 Tjele, Denmark

**Keywords:** transcriptome, colitis, pig, dextran sodium sulfate

## Abstract

Dextran sodium sulfate (DSS) was used to induce ulcerative colitis in a porcine model to investigate the transcriptional responses in inflamed colonic tissue. Eleven pigs were divided into two groups, of which five pigs were administered an oral dose of DSS daily for five days, whereas six non-treated pigs served as a control group. Differences in transcript expression between treated and control pigs identified 425 down-regulated and 780 up-regulated mRNAs in response to DSS treatment. Fifty-nine differentially expressed miRNAs were also identified, comprising 20 up-regulated and 39 down-regulated miRNAs. The top enrichment KEGG pathways for the up-regulated genes were breast cancer (ssc05224), gastric cancer (ssc05226), focal adhesion (ssc04510), and the PI3K-Akt signaling pathway (ssc04151). The top gene ontology terms for the up-regulated genes were blood vessel development (GO:0001568), extracellular matrix and external encapsulating structure (GO:0031012). Protein–protein interaction network analysis identified three hub genes, including *LOXL1*, *MFAP2*, and *FSTL3*. Seventeen high-confidence miRNA-mRNA pairs were recognized, and two genes (*CD101* and *AVL9*) have been confirmed as targets for ssc-miR-24-3p by dual-luciferase reporter assay. Our study provides a better understanding of the key roles of mRNAs and miRNAs in regulating DSS-induced colitis in pigs and defines sets of coding and non-coding RNA transcripts, which may serve as intervention targets or biomarkers for ulcerative colitis.

## Simple Summary

This study employed a porcine model of dextran sodium sulfate (DSS)-induced ulcerative colitis to investigate mRNA and miRNA alterations in inflamed colon tissue. Eleven pigs were assigned to a DSS-treated group (*n* = 5) or a control group (*n* = 6). Compared with controls, DSS treatment resulted in 425 down-regulated and 780 up-regulated mRNAs, plus 59 differentially expressed miRNAs (20 up-regulated and 39 down-regulated). Enrichment analysis indicated that the most prominently affected KEGG pathways and GO terms for up-regulated genes were breast cancer, focal adhesion, PI3K-Akt signaling, extracellular matrix, external encapsulating structure and blood vessel development. Three hub genes (*LOXL1*, *MFAP2*, *FSTL3*) and 17 high-confidence miRNA-mRNA pairs were identified, of which miR-24-3p targeting the two genes *CD101* and *AVL9* was validated by dual-luciferase reporter assays. These findings elucidate key mRNA and miRNA regulatory mechanisms in DSS-induced porcine colitis and offer potential biomarkers and therapeutic targets for ulcerative colitis.

## 1. Introduction

Inflammatory bowel disease (IBD), encompassing ulcerative colitis (UC) and Crohn’s disease, is characterized by chronic inflammation of the gastrointestinal tract and an elevated risk of colorectal cancer [1]. The etiology is multifactorial, involving genetic predisposition, gut dysbiosis, environmental factors, and individual lifestyles [2,3,4]. Clinical manifestations of UC are heterogeneous and may include diarrhea, rectal bleeding, cramp-like abdominal pain, urgency and frequent bowel movements, and various extra-intestinal manifestations such as peripheral arthritis. UC may also give rise to serious complications including autoimmune reactions, toxic dilatation, enteritis, and the development of polyps and colon cancer [5]. A thorough understanding of the specific pathophysiology and molecular regulatory mechanisms underlying UC is, therefore, essential for disease prevention and the development of effective therapeutic interventions.

Oral administration of dextran sodium sulfate (DSS), a sulfated polysaccharide, disrupts the intestinal epithelium lining of the colon, allowing luminal bacteria and associated antigens to penetrate the mucosa, thereby triggering intestinal inflammation [6]. Since its introduction in 1985 [7], DSS-induced colitis in animal models, particularly the mouse, has become a widely used tool in UC research owing to its simplicity, cost-effectiveness, and reproducibility. Murine models mimic many critical aspects of human UC but do not fully reproduce all clinical manifestations. Nevertheless, DSS models are highly adaptable, allowing the induction of acute, chronic or relapsing colitis by varying the molecular weight and concentration of DSS, the frequency and duration of treatment, the genetic background of the mouse strain, and the composition of the gut microbiota [8]. In addition to the widely used rodent models, the domestic pig has emerged as a reliable DSS-induced colitis model. Pigs are omnivorous, with remarkable anatomical and physiological similarities to humans in their gastrointestinal tract, including a villus architecture and the cellular composition of the intestinal epithelium [9]. Porcine experimental models have proven valuable for evaluating preventive or therapeutic strategies, including supplementation with amino acids, fatty acids or soy-derived peptides [10,11,12,13]; the effect of dietary meat consumption [14,15]; probiotic administration [16]; and gut microbiota–host interactions in the context of resistance to inflammatory bowel disease [17,18].

A deeper understanding of the fundamental molecular mechanisms and pathophysiology of UC is still needed to facilitate the development of novel diagnostic and therapeutic approaches. To address this, we employed high-throughput transcriptomic analysis to profile coding and non-coding gene expression in the inflamed colon of DSS-treated pigs. Analysis of differentially expressed (DE) mRNAs and miRNAs, combined with enrichment analysis of gene ontology (GO) terms and Kyoto Encyclopedia of Genes and Genomes (KEGG) pathways, revealed several biological processes associated with DSS-induced UC, most notably vascular development and cancer-related processes. These data enhance our understanding of gene expression responses in the colon of a DSS-treated porcine model and highlight the involvement of angiogenic factors in driving the pathogenesis of ulcerative colitis [19].

## 2. Materials and Methods

### 2.1. Ethics Statement

The animal experiments were performed previously as part of an earlier published study [14], from which all biological samples used in the present manuscript were obtained. Following sample collection, all colon tissue samples (*n* = 12) were stored at −80 °C, so we did not perform any experiments on animals in this current study. Specifically, all samples were collected under the license (2016-15-0201-01033) obtained from the Danish Animal Experimentation Inspectorate, Ministry of Food, Agriculture and Fisheries. Throughout the experiment, animal health and welfare were monitored daily, and appropriate measures were taken to minimize pain, distress, and suffering in accordance with the approved experimental protocol in the previous study [14]. All animal procedures were performed in compliance with the permit of Danish laws and regulations for the humane care and use of animals in research (The Danish Ministry of Justice, Animal Testing Act no. 1306 of 23 November 2007). All details of animal care and study design have been described carefully in the previous study [14]. Unfortunately, one colon tissue sample in the DSS group was lost, so only 11 samples, including five samples in the DSS group and six samples in the control group, were used to sequence in our study.

### 2.2. RNA Isolation, Library Preparation, and Sequencing

After sample collection from the previous study [14], we immediately isolated RNA for sequencing in our study. The generated sequencing data were used for subsequent analysis. RNA quantity and quality were assessed using a NanoPhotometer^®^ spectrophotometer (IMPLEN, Westlake Village, CA, USA), an Agilent Bioanalyzer 2100 system (Agilent Technologies, Santa Clara, CA, USA), and 1% agarose gels. Ribosomal RNA was depleted using the Epicentre Ribo-zeroTM rRNA Removal Kit (Epicentre, Madison, WI, USA). Sequencing libraries were prepared from rRNA-depleted RNA using the NEBNext^®^ UltraTM Directional RNA Library Prep Kit for Illumina^®^ (NEB, Ipswich, MA, USA) according to the manufacturer’s instructions. cDNA library was subsequently generated and sequenced on the Illumina HiSeq platform to generate paired-end reads.

### 2.3. Small RNA Isolation, Library Preparation, and Sequencing

A total amount of 3 μg total RNA per sample was used as input material for the small RNA library construction. Sequencing libraries were prepared using the NEBNext^®^ Multiplex Small RNA Library Prep Set for Illumina^®^ (NEB, Ipswich, MA, USA) following the manufacturer’s instructions. Briefly, the NEB 3’ SR Adaptor was ligated directly to the 3’ end of miRNA, siRNA and piRNA molecules. Following 3’ ligation, the SR RT Primer was hybridized to excess free 3’ SR Adaptor, converting the single-stranded DNA adaptor into a double-stranded DNA molecule. First-strand cDNA synthesis was performed using M-MuLV Reverse Transcriptase (RNase H). PCR amplification products were purified by electrophoresis on an 8% polyacrylamide gel (100 V, 80 min). DNA fragments of 140–160 bp were excised, recovered, and dissolved in 8 μL elution buffer. Library quality was assessed using the Agilent Bioanalyzer 2100 system with DNA High Sensitivity Chips.

Clean sRNA reads were aligned to the miRBase20.0 database using the modified *mirdeep2* (version 0.1.3) software [20] to identify known and novel miRNAs. Small RNA tags were additionally mapped to the *RepeatMasker* (version open-4.0.3) and Rfam database to ensure accurate and unique annotation. The following annotation priority order was applied: known miRNA > rRNA > tRNA > snRNA > snoRNA > repeat > gene > NAT-siRNA > gene > novel miRNA > tasiRNA. The proportion of rRNA reads served as a quality indicator for each sample.

### 2.4. Quality Control and Differentially Expressed Gene Analysis

Raw data quality was assessed by calculating Q20, Q30, and GC content using FastQC (version 0.11.9) [21]. Clean reads of sRNA and mRNA were obtained by removing raw reads with adapters, poly-N, low-quality reads, and too-short reads using *fastp* (version 0.23.1) [22]. Clean mRNA reads were aligned to the reference genome *Sscrofa11.1* using *Hisat2* (version 2.0.4) [23]. *Samtools* (version 1.20) [24] was used to index BAM files and evaluate mapping quality. Transcript assembly and quantification were performed using StringTie (version 3.0.3) [25], and DE transcripts were identified using the R package *Ballgown* (version 2.26.0) [25]. The R package *BiomaRt* (version 2.48.3) [26] with *getBM* function to get *sscrofa_gene_ensembl* information from the reference genome *Sscrofa11.1*. For miRNAs, differential expression analysis was conducted with DESeq2 [27] based on raw read counts. Multiple hypothesis testing correction was performed using Benjamini–Hochberg method. Transcript and miRNAs with |Log_2_(Fold Change)| ≥ 1 and an adjusted *p*-value ≤ 0.05 were defined as differentially expressed. Genome-wide annotations for all DE genes were analyzed by pig database *org.Ss.eg.db* (version 3.8.2). The GO terms and KEGG pathways of all DE genes were enriched using *clusterProfiler* (version 4.1) [28].

### 2.5. Protein–Protein Interaction Analysis of Differentially Expressed Genes

Protein–protein interactions (PPIs) for DE genes were predicted using the STRING database (https://string-db.org/), with a minimum combined score of 0.4. Network centrality parameters of degree, betweenness, and eigenvector were calculated using the CentiScaPe plugin (version 2.2) [29] in Cystoscope software (version 3.10.2). Nodes exceeding the mean value for each parameter were selected, and their overlap across all three parameters was visualized using Venny 2.1 [30]. Hub genes were identified by using the CytoHubba plugin (version 0.1) in Cytoscape 3.10.4 [31], wherein the top 10 genes ranked by degree were designated as hub genes. PPI networks were visualized in Cystoscope, with node color indicating the degree of connectivity.

### 2.6. Prediction of Candidate miRNA-mRNA Regulatory Interactions

To identify putative miRNA-mRNA regulatory interactions associated with colitis, miRNA target prediction was performed using three complementary computational tools: miRanda (version 3.3a) [32], RNAhybrid (version 2.1.2) [33], and RNA22 (version 2.0) [34]. Only DE miRNAs and mRNAs exhibiting significant negative expression correlation (*r* < −0.6, *p* < 0.05) were included as input. Full-length *Sus scrofa* transcript sequences, including 5’-untranslated regions (5’UTRs), coding sequences (CDS), and 3’-untranslated regions (3’-UTRs), were retrieved from the Ensembl *Sscrofa11.1* reference genome and applied consistently across all tools. miRanda was applied using an alignment score threshold of 140 and a minimum free energy (MFE) cutoff of −20 kcal/mol. RNAhybrid was run in compact mode with an MFE cutoff of −20 kcal/mol. RNA22 was applied with default parameters, allowing detection of seed-independent and non-canonical binding sites. For all tools, predicted binding sites were assigned to transcript regions (5’UTR, CDS, or 3’UTR) based on CDS annotations. Predicted miRNA–mRNA interactions were filtered to retain only pairs supported by inverse expression correlation and with binding sites located within the 3’-UTR. High-confidence interactions were defined as those predicted by all three prediction tools. Overlap among the prediction methods was visualized using the VennDiagram package (version 1.7.3).

### 2.7. Plasmid Construction, Transfection and Dual Luciferase

Mutated and non-mutated DNA fragments containing the ssc-miR-24-3p target site in the 3’UTR of porcine CD101 molecule gene (*CD101*) and AVL9 cell migration-associated gene (*AVL9*) were cloned into the NheI-SalI sites of the pmirGLO vector. Four pmirGLO vectors, including CD101-MUT, CD101-WT, AVL9-MUT, and AVL9-WT, were constructed. The sequence of the mimic for ssc-miR-24-3p is UGGCUCAGUUCAGCAGGAACAG, and its control is UUUGUACUACACAAAAGUACUG. All the sequences were synthesized by GENCEFE Biotech (Wuxi, China). The pmirGLO vectors, mimics, and mimics NC were transfected with Lipofectamine 3000 (Invitrogen, ThermoFisher, Waltham, MA, USA) to HEK293T cells and incubated with 5% CO_2_ at 37 °C.

Dual-luciferase reporter assay kit (Vazyme Co. Ltd., Nanjing, China) was used to test the interaction of ssc-miR-24-3p and *CD101* and *AVL9* genes. The results were calculated as the ratios of firefly to renilla luciferase activities in three independent replicates. Student’s *t*-test was used for the significance test, and *p* < 0.05 was considered statistically significant.

## 3. Results

### 3.1. Quality Control and Alignment for the RNA Sequencing Data

RNA-seq was used to identify and quantify the expression levels of genes regulated in response to DSS treatment in pigs. On average, 78,877,050 and 79,739,666 raw reads were generated from the colon tissue of the CON and DSS groups, respectively (Table S1). After filtering low-quality reads, too-short reads, and reads containing adaptors and redundant Ns, average clean read counts of 67,380,251 and 78,773,351 were obtained for the CON and DSS groups in the mRNA sequencing data, respectively (Table S1). Approximately 73.59% and 75.59% of the clean reads from the CON and DSS groups, respectively, were mapped to the porcine reference genome (version Sscrofa11.1) as properly paired reads (Table S2). Reads that were unmapped or mapped to multiple locations were excluded.

To characterize miRNA expression profiles in the colon between the two groups, small RNA libraries were constructed from the colonic tissue of both the CON and DSS-treated pigs. On average, 21,144,913 and 21,095,098 raw reads were generated in the CON and DSS groups, respectively (Table S3). An average of 20,364,662 and 19,629,310 clean reads were obtained for the CON and DSS groups, respectively, in the miRNA sequencing data after removing reads longer than 35 bp and reads shorter than 16 bp. The mapping rate of clean sRNA reads to the porcine reference genome ranged from 52.74% to 78.14%, with more than 50% of reads successfully mapped (Table S4).

### 3.2. Identification of Highly Abundant and Differentially Expressed Transcripts

**Figure 1:**
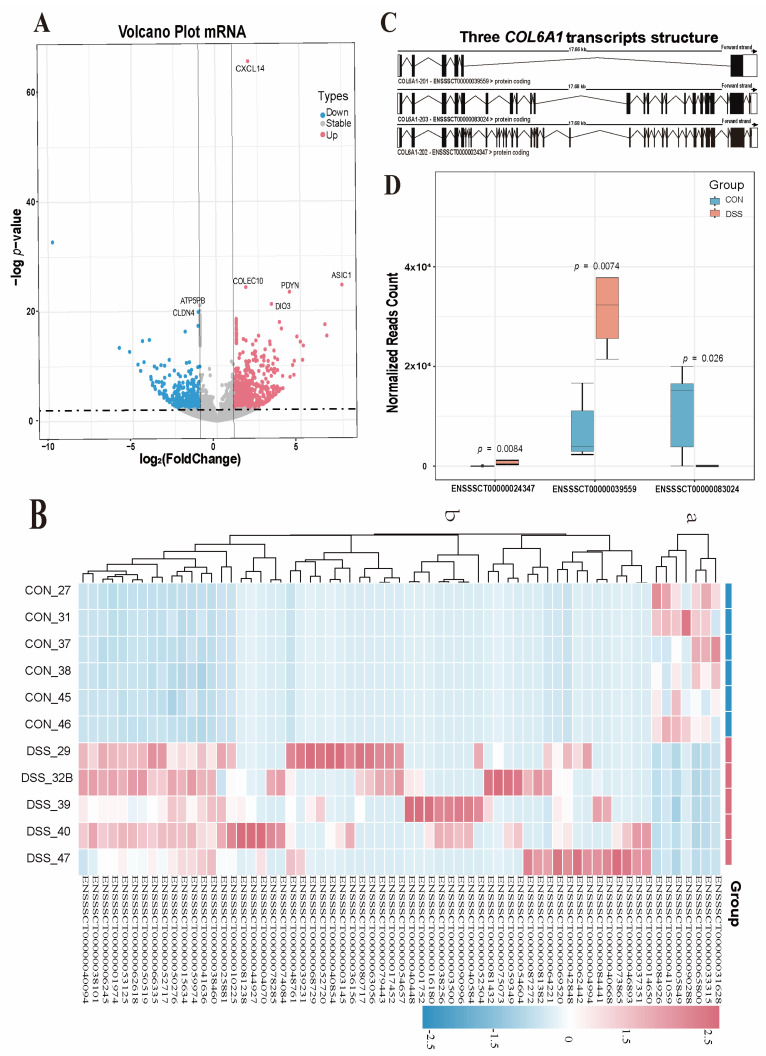
The differentially expressed transcripts. (**A**) Volcano plot for the transcripts. (**B**) Heatmap for the differentially expressed transcripts (−log *p*-value > 13 and |Log_2_FC| > 1). (**C**) The three transcripts’ structure for collagen type VI alpha 1 chain gene (*COL6A1*). (**D**) The expression of *COL6A1*’s three transcripts between DSS group and CON group. a: down-regulated transcripts, b: up-regulated transcripts.

After the low-abundance transcripts were filtered, 22,779 annotated transcripts and 20,700 novel-predicted transcripts starting with the name ‘MSTRG’ were obtained (Supplemental File S1). Here, we identified 425 down-regulated and 780 up-regulated transcripts according to the thresholds of adjusted *p*-value < 0.05 and |Log_2_FC| > 1 (Figure 1A). Detailed information on all DE transcripts is provided in Supplementary File S3. The most significant up-regulated transcript was C-X-C motif chemokine ligand (*CXCL14*), followed by acid-sensing ion channel subunit 1 (*ASIC1*). Among the down-regulated transcripts, the most significant was an ensemble-annotated novel transcript (ENSSSCT00000084926), followed by proteasome 20S subunit beta 10 (*PSMB10*) (Table 1). A heatmap based on Z-score-normalized read counts was constructed to visualize DE transcript expression between the DSS and CON groups. Transcripts with Log_10_(*p*-value) > 13 and |Log_2_FC| > 1 clustered into two distinct groups corresponding to up- and down-regulated transcripts (Figure 1B). Additionally, six DE genes, including collagen type VI alpha 1 chain gene (*COL6A1*), protocadherin gamma subfamily A 4 (*PCDHGA4*), Rho guanine nucleotide exchange factor 28 gene (*ARHGEF28*), sorting nexin 4 gene (*SNX4*), collagen type XVIII alpha 1 chain gene (*COL18A1*), and ENSSSCG00000026996, exhibited transcript-specific differential expression (Supplemental File S2). For example, *COL6A1* produces three distinct transcripts (Figure 1C): ENSSSCT00000024347 and ENSSSCT00000039559 were significantly up-regulated in the DSS group, whereas ENSSSCT00000083024 was significantly down-regulated (Figure 1D).

### 3.3. Functional Enrichment of Differentially Expressed Transcripts

**Figure 2:**
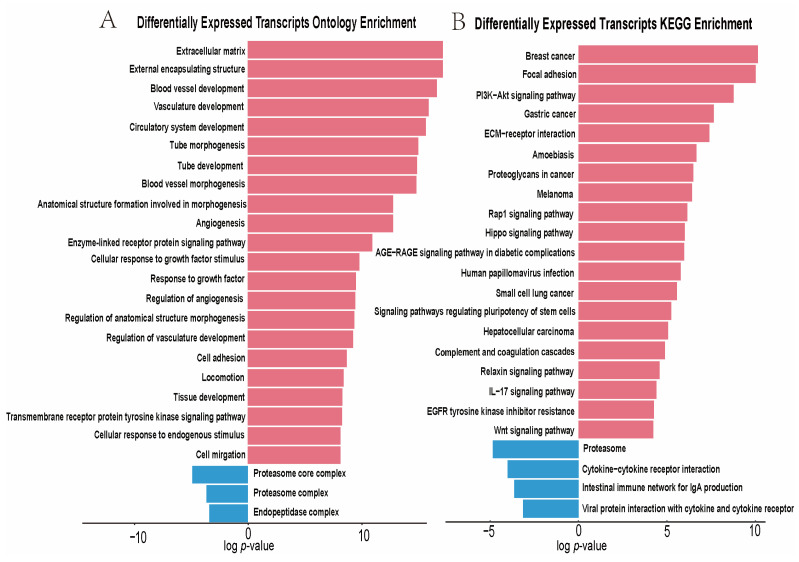
Functional enrichment analysis. (**A**) The gene ontology (GO) term enrichment for the up-regulated transcript (adjusted *p*-value < 0.000001) and the down-regulated transcript analysis (adjusted *p*-value < 0.05). (**B**) Kyoto Encyclopedia of Genes and Genomes (KEGG) enrichment for the up-regulated (adjusted *p*-value < 0.0001) and down-regulated transcript analysis (adjusted *p*-value < 0.05).

In total, 97.70% of the 780 up-regulated transcripts were successfully mapped to the porcine annotation database org.Ss.eg.db. Enrichment analysis identified 424 significant GO terms (adjusted *p*-value < 0.05) comprising 391 biological process (BP), 10 cellular component (CC), and 23 molecular functions (MF) (Supplemental File S4). The most significantly enriched GO terms were external encapsulating structure (GO:0030312), extracellular matrix (GO:0031012) and blood vessel development (GO:0001568), associated with 31, 31, and 38 genes, respectively (Figure 2A, Supplemental File S4). A total of 281 significant KEGG pathways were enriched for the up-regulated transcripts (adjusted *p*-value < 0.05). Among the most enriched KEGG pathways were focal adhesion (ssc04510), the PI3K-Akt signaling pathway (ssc04151), ECM–receptor interaction and amoebiasis, involving 28, 36, 16 and 16 up-regulated genes, respectively (Figure 2B, Supplemental File S5). Seven genes were commonly enriched across all four KEGG pathways (Figure S1A, Table S5). Notably, pathways underlying several cancer types were observed, such as gastric cancer, melanoma, and small-cell lung cancer, possibly indicating cell proliferation and tissue growth. Likewise, 97.53% of the 425 down-regulated transcripts were mapped, and three significant GO terms were identified: proteasome core complex (GO:0005839), proteasome complex (GO:0000502), and endopeptidase complex (GO:1905369) (Figure 2A, Supplemental File S4). Significant KEGG pathways for the down-regulated genes included the proteasome (ssc03050), cytokine–cytokine receptor interaction (ssc04060), and the intestinal immune network for IgA production (ssc04672), involving 7, 15, and 6 down-regulated genes, respectively (Figure 2B, Supplemental File S5). Four genes, including C-C motif chemokine ligand 28 gene (*CCL28*), TNF superfamily member 13 gene (*TNFSF13*), C-C motif chemokine receptor 10 gene (*CCR10*) and TNF receptor superfamily member 17 (*TNFRSF17*), were commonly enriched across all three down-regulated KEGG pathways (Figure S1B, Table S5). These results demonstrate that up-regulated and down-regulated DE genes are enriched in distinct KEGG pathways.

### 3.4. Hub Gene Identification Through Protein–Protein Interaction (PPI) Network Analysis

**Figure 3:**
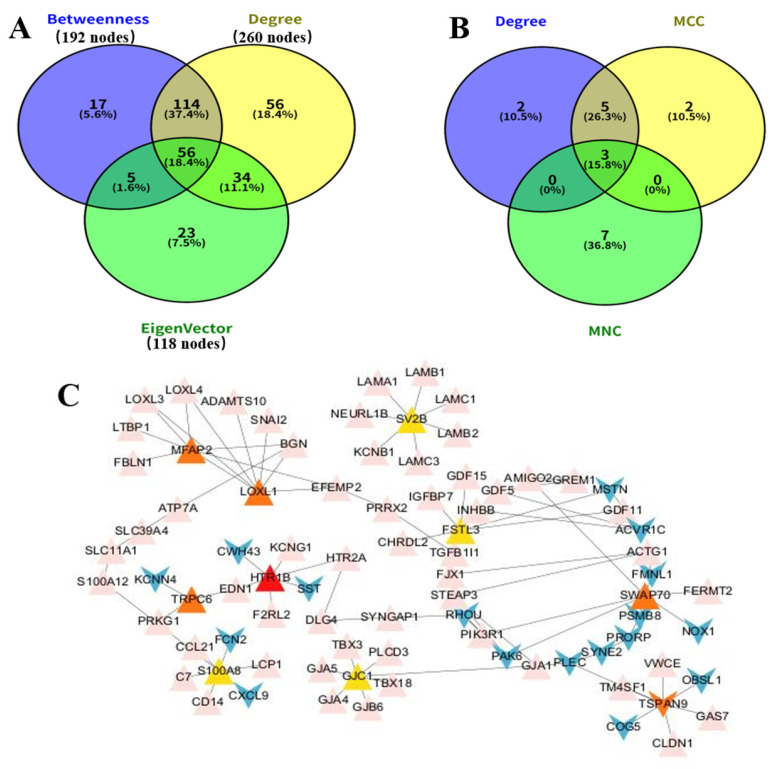
Protein–protein network (PPI) analysis of the differentially expressed genes (DEGs). (**A**) Venn diagram showing nodes with the values above the average score of Degree, Betweenness, and eigenvector. (**B**) Venn diagram showing the top ten hub genes using the MCC, Degree, and MNC algorithms. (**C**) PPI network for the hub genes based on the Degree algorithms. Upward triangle represents the up-regulated genes, while downward triangle represents the down-regulated genes. Node color graduates from yellow to red based on the degree of connectivity from low to high.

A PPI network was constructed to explore the interaction relationships among the DEGs. In total, 869 nodes and 2989 edges were identified by using the STRING database, with the threshold of a combined interaction score of 0.4. A core high-centrality subnetwork was then derived through a three-step filtering process: first, 260 nodes with degree values exceeding the network mean of 2.85 were retained; second, 192 nodes with betweenness values above the mean of 733.11 were identified as network bottlenecks; and third, 118 nodes with eigenvector values exceeding the mean of 0.001 were selected. The intersection of these three criteria yielded 160 high-centrality genes, representing 18.4% of the original PPI network (Figure 3A), with the refined network comprising 260 nodes and 280 edges. Hub gene identification was further performed using the cytoHubba plugin with three algorithms: MCC, Degree, and MNC. Three up-regulated genes, including lysyl oxidase-like 1 (*LOXL1*), microfibril-associated protein 2 (*MFAP2*), and follistatin-like 3 (*FSTL3*), were consistently ranked within the top ten across all three algorithms and were, therefore, designated as hub genes (Table 2, Figure 3B). The PPI network with high-centrality values was constructed based on the Degree algorithm (Figure 3C). Five hub genes with similar degree scores of eight each interacted with at least six other genes; for example, the up-regulated gene *LOXL1* was connected to seven other up-regulated genes, including *ASAMTS*10, *EFEMP2*, *LOXL3*, *LOXL4*, *LTBP1*, *MFAP2* and *FBLN1*.

### 3.5. Identification of Highly Abundant and Differentially Expressed miRNAs

**Figure 4:**
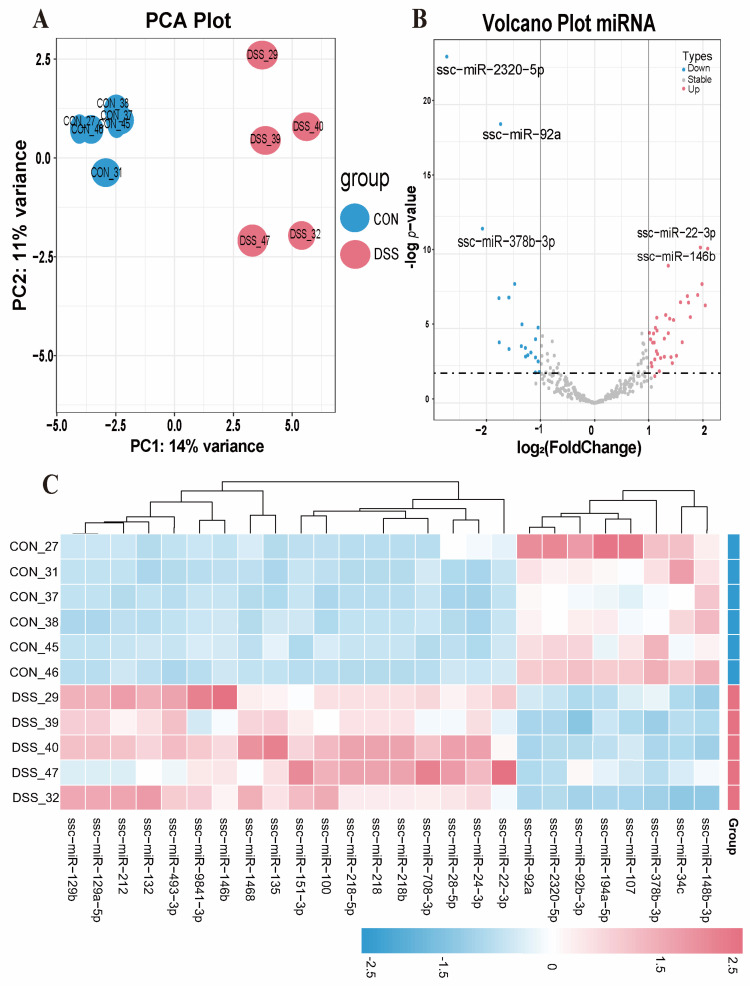
Differentially expressed miRNAs. (**A**) Principal component analysis (PCA) of the miRNAs. (**B**) Volcano plot for the differentially expressed miRNAs. (**C**) Heatmap for the differentially expressed miRNAs (adjusted *p*-value < 0.05 and |log_2_FoldChange| > 1).

After low-abundance miRNAs were filtered, a total of 296 miRNAs were retained (Supplemental File S6). Principal component analysis (PCA) demonstrated distinct miRNA expression profiles between the CON and DSS groups (Figure 4A). A total of 59 DE miRNAs were identified, including 20 up-regulated and 39 down-regulated miRNAs (Figure 4B). The two most highly up-regulated miRNAs were ssc-miR-146b and ssc-miR-22-3p, while the two most down-regulated were ssc-miR-2320-5p and ssc-miR-92a (Table 3). Hierarchical clustering of the miRNA expression profiles displayed two different clusters corresponding to DSS-treated and non-DSS-treated pigs (Figure 4C).

### 3.6. Identification of Candidate miRNA-mRNA Regulatory Interactions

High-confidence miRNA-mRNA regulatory interactions were identified by calculating Spearman correlation coefficients between 442 DE transcripts and 59 miRNAs based on normalized read counts. In total, 19,111 positively correlated pairs (*r* > 0.6, *p* < 0.05) and 14,597 negatively correlated pairs (*r* < −0.6, *p* < 0.05) were identified (Supplemental File S7). Target prediction was performed using miRanda, RNAhybrid, and RNA22, with further filtering for pairs exhibiting inverse expression patterns and 3′UTR binding sites. RNAhybrid predicted the largest number of candidate interactions (219 miRNA–mRNA pairs), followed by RNA22 (106 pairs) and miRanda (20 pairs). Notably, no interactions were predicted exclusively by miRanda; all miRanda-supported pairs were also identified by at least one of the other tools. This indicates substantial concordance among the prediction methods. The overlap among prediction tools was visualized using a Venn diagram (Figure S2). Integration of the prediction tools from miRanda, RNAhybrid and RNA22 yielded 17 high-confidence miRNA–mRNA interactions (Table 4). These consensus interactions, supported by multiple prediction algorithms and inverse expression patterns, represent biologically relevant miRNA-mediated regulatory relationships in porcine colitis.

### 3.7. Verification of ssc-miR-24-3p Targeting CD101 and AVL9 Genes

**Figure 5:**
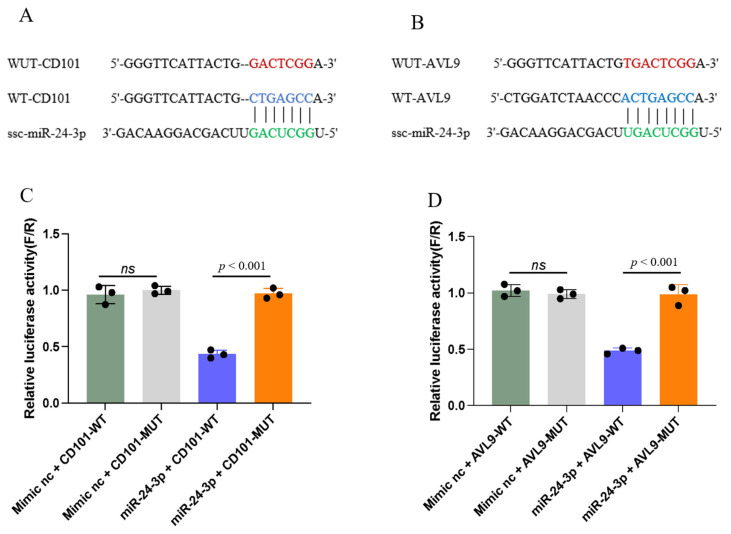
Verification of ssc-miR-24-3p targeting *CD101* and *AVL9* genes. (**A**) Target site for *CD101*. (**B**) Target site for *AVL9*. (**C**) Luciferase activity reporter when transfected with CD101-MUT, CD101-WT, ssc-miR-24-3p mimic and NC. (**D**) Luciferase activity reporter when transfected with AVL9-MUT, AVL9-WT, ssc-miR-24-3p mimic and NC. F/R means the ratio of luciferase activity of firefly and renilla. Each group has three independent replicates. Student’s *t*-test was used for the significance test, and *p* < 0.05 was considered as statistically significant; ns means no statistical significance.

To verify the 17 predicted high-confidence miRNA–mRNA interactions, miR-24-3p and two genes (*CD101* and *AVL9*) were chosen for dual-luciferase reporter experiments. Co-transfection with miR-24-3p mimics and CD101-WT or AVL9-WT markedly inhibited F/R luciferase activity, while co-transfection of miR-24-3p mimics with CD101-MUT or AVL9-MUT exerted no inhibitory effect on F/R luciferase activity, which indicates that miR-24-3p binds specifically to *CD101* and *AVL9* (Figure 5).

## 4. Discussion

Due to their comparable gastrointestinal anatomy and physiology, as well as similar nutrient requirements and immune responses, pigs represent an excellent animal model for studying human UC [14,35,36]. Although a limited number of studies have employed microarrays or RNA-seq to identify coding gene differences in DSS-induced porcine colitis models [8,9,10,11], comprehensive transcript–miRNA regulatory networks have not yet been fully characterized in pigs. In the present study, high-throughput transcriptomic analysis identified 1205 DE transcripts and 59 DE miRNAs between DSS-induced porcine colitis and control groups. Through functional enrichment analysis, PPI network construction and miRNA-mRNA interaction prediction, three hub genes and 17 high-confidence miRNA-mRNA pairs were predicted. Two target genes of ssc-miR-24-3p have been confirmed by dual-luciferase reporter assay, providing preliminary insights into the molecular mechanism for colitis. Overall, our data suggests that blood vessel development and cancer-related pathways are activated in ulcerative colitis induced by DSS treatment.

Among all DE transcripts, *CXCL14* showed the greatest up-regulation in the DSS-induced group, which is consistent with Zeng et al.’s research [37] but inconsistent with Kezhi et al.’s research [38], who reported that *CXCL14* was remarkably reduced in colorectal carcinoma. *CXCL14* was initially identified in 1999, and its porcine amino acid sequences are 94% identical to the human counterpart [39]. It plays modulatory roles in immune and inflammatory responses [40] and exhibits anti-colorectal cancer activity [41]. Predominantly expressed by intestinal lamina propria fibroblasts, *CXCL14* has been proposed as a candidate target for anti-fibrotic and tumor immunotherapies [42]. As chronic colitis, particularly IBD and Crohn’s disease (CD), is a major cause of intestinal fibrosis, anti-fibrotic interventions are crucial for preventing colitis [39]. Consistent with a previous study using qRT-PCR [14], the prostaglandin-endoperoxide synthase2 (*PTGS2*) gene and interleukin 6 (*IL-6*) gene were also highly expressed in the DSS-induced group according to mRNA-seq. The *PTGS2* gene has been proposed as a candidate target for alleviating intestinal inflammation, preventing fibrosis, and improving colitis prognosis by catalyzing the synthesis of prostaglandin E2 (PGE2), predominantly PGE_2_ [43]. In addition, the pro-angiogenic and pro-inflammatory genes, including *CXCL8* (*IL-8*), *IL-6*, and *IL-1A*, which have been implicated in the initiation, progression, and tissue injury of intestinal inflammation [19,44], were also up-regulated in the DSS-induced group. In addition, we observed that the three transcripts from the *COL6A1* gene were differentially expressed between the two groups. These discrepancies may have arisen from the diverse transcript structures and variations in quantification models [45].

Enrichment analysis revealed a strong over-representation of GO associated with vascular development and angiogenesis, including blood vessel development, tube morphogenesis, angiogenesis, and the regulation of vascular development. This finding is consistent with the previously reported involvement of angiogenesis in IBD, where it serves as a hallmark of chronic mucosal inflammation [46]. Angiogenesis contributes to IBD pathogenesis by promoting the infiltration of immune cells and inflammatory mediators into the intestinal mucosa, thereby facilitating barrier disruption and tissue damage [19]. Notably, these angiogenic changes occurred alongside the down-regulation of IFNG, suggesting that IFN-γ-independent mechanisms, potentially driven by innate immune cytokines, predominate in this acute DSS-induced colitis model. Among angiogenic growth factors, *VEGFC*, *VEGFR-2* (also known *KDR*) and *FGF2* were markedly elevated. *VEGFC*, the only up-regulated member of the VEGF family in our dataset, has been implicated in promoting lymph angiogenesis and mucosal remodeling in IBD. Although enhanced lymphatic drainage may aid in resolving inflammation [47], aberrant or excessive lymph angiogenesis can exacerbate disease pathology [48], which highlights its context-dependent effects. Additionally, increased expression of angiopoietins, including *ANGPT2*, *ANGPTL2*, and *ANGPTL4*, could regulate vascular remodeling and endothelial permeability. *ANGPT2*, in particular, is known to destabilize endothelial junctions and facilitate inflammation-induced angiogenesis permeability [19]. The up-regulation of *MADCAM1*, a gut-specific endothelial adhesion molecule involved in lymphocyte recruitment [46], further supports sustained immune cell infiltration into the inflamed mucosa. The prominence of angiogenesis-related pathways in our dataset supports the suitability of the porcine model for IBD and aligns with previous reports in both human patients and rodent models. Furthermore, transcriptome sequencing enables the annotation and discovery of novel transcripts and provides refined information on gene structure and chromosomal localization [49].

The PI3K/AKT signaling pathway plays an important role in the development and progression of UC [50,51]. Suppression of PI3K/Akt/NF-κB signaling has been shown to attenuate DSS-induced colitis in mice by reducing the production of pro-inflammatory mediators [52,53]. Consistent with previous studies [54,55], which reported that hub DE genes in patients with colitis-associated colon cancer were enriched in this pathway, 36 up-regulated genes were also enriched in the PI3K/AKT pathway. Notably, 22 up-regulated genes, such as AKT serine/threonine kinase 3 gene (*AKT3*), insulin-like growth factor 1 (*IGF1*), and the secreted phosphoprotein 1 gene (*SPP1*), were commonly enriched in both the PI3K/AKT pathway and the focal adhesion pathway. Focal adhesions are dynamic connection complexes between cells and the extracellular matrix (ECM) that regulate cell adhesion, migration, proliferation, and differentiation. Previous studies have demonstrated that acesulfame potassium can trigger IBD via inhibition of the focal adhesion pathway [56]. In addition, arctigenin [57] and DIREN [58] could inhibit the focal adhesion pathway to promote mucosal healing in ulcerative colitis patients or to mitigate DSS-induced colitis in mice.

PPI network analysis integrating Degree, Betweenness, and eigenvector centrality algorithms identified three up-regulated hub genes, including *LOXL1*, *MFAP2*, and *FSTL3*. As a member of the lysyl oxidase family, *LOXL1* is involved in the cross-linking of collagen and elastin within the ECM [59], and its aberrant expression has been associated with tumor progression and metastasis [60]. *MFAP2*, a structural component of the ECM, is primarily involved in microfibril assembly and stability; and its elevated expression may promote ECM remodeling dysregulation and compromise the integrity of the intestinal epithelial barrier [61]. As a secreted glycoprotein, *FSTL3* participates in the regulation of cell survival, proliferation, differentiation and migration [62] and may exert a pivotal role in multiple inflammatory diseases. Consistent with its known function in inflammatory processes, the elevated expression of *FSTL3* in DSS-induced groups in our study suggests that it may amplify intestinal inflammation by modulating immune cell function [63]. The identification of these three hub genes provides key targets for further in-depth investigation of the molecular regulatory mechanism underlying colitis. However, their specific regulatory pathways require validation through in vitro experiments and in vivo animal studies.

Various miRNAs have been identified as important biomarkers for inflammation, immune regulation, and IBD [64,65,66]. For instance, miR-146b has been reported to improve epithelial barrier function and thereby alleviate intestinal inflammation [67], as well as to target intestinal macrophages to promote mucosal regeneration in a murine colitis model [68]. Consistent with previous studies [69], miR-24-3p was elevated in the ulcerative colitis patients and up-regulated in the DSS-induced groups in the present study. Loss of miR-24-3p may contribute to epithelial cell apoptosis and exacerbate intestinal inflammation [70]. In addition, dual-luciferase reporter assays validated ssc-miR-24-3p as an upstream regulator of *AVL9*, whose expression was suppressed in the DSS-induced group. Clinically, accumulated evidence indicates that high *AVL9* expression is a marker of unfavorable prognosis for colorectal carcinoma patients [71]. Researchers also have found that *CD101* exerted an inhibitory effect on the expansion of colitogenic T cells [72], and the loss of *CD101* exacerbates DSS-induced colitis [73]. Dual-luciferase reporter assays also validated that ssc-miR-24-3p could suppress *CD101* gene expression.

Despite the high degree of genomic homology between pigs and humans, notable differences in gene expression patterns exist between the two species. Nonetheless, the present findings provide a useful transcriptomic foundation for modeling human colonic diseases in pigs. However, to fully realize the translational potential of the porcine colitis model for human disease, further investigation from a multi-omics perspective is warranted. Our study also has several limitations. First, the identification of differential molecules and regulatory relationships was based solely on bioinformatics analysis; all predicted miRNA-mRNA pairs and the functions of hub genes require experimental validation through in vitro dual-luciferase reporter assays and in vivo animal experiments. Second, the biological functions of transcripts (e.g., ENSSSCT00000084926) may represent non-coding RNAs, alternatively spliced isoforms, or lineage-specific unannotated coding genes, and their regulatory roles in colitis remain to be elucidated. Third, the limited sample size reduced the statistical power to detect subtle correlations between gene expression and clinical parameters. Finally, this study focused exclusively on the transcriptome and miRNA levels and did not integrate complementary omics data such as proteomics, which limits a comprehensive understanding of the molecular regulatory network underlying colitis.

## 5. Conclusions

Our study provided preliminary computational insights into the molecular features underlying DSS-induced colitis in a porcine model at the mRNA and miRNA levels. The 22 up-regulated genes were enriched in both the focal adhesion and the PI3K-Akt signaling pathway in DSS-induced pigs. PPI network analysis identified three hub genes, including *LOXL1*, *MFAP2*, and *FSTL3*. Seventeen high-confidence miRNA-mRNA pairs were recognized; for instance, miR-24-3p may target *AVL9*, and ssc-miR-132 may target *CCL28*. These findings provide preliminary computational evidence that may contribute to a better understanding of the molecular landscape of DSS-induced colitis and suggest candidate pathways and gene targets for future experimental validation as potential diagnostic markers or therapeutic targets.

## Figures and Tables

**Table 1:** Top five up- and down-regulated abundant and differentially expressed transcripts.

ENSEMBL	Name	Chr	log_2_FoldChange	Adjusted *p*-Value	Up/Down	−log *p*-Value
ENSSSCT00000015634	*CXCL14*	2	1.955395	1.07 × 10^−61^	Up	65.58624816
ENSSSCT00000038460	*ASIC1*	5	7.481973	1.03 × 10^−21^	Up	24.69895858
ENSSSCT00000041636	*COLEC10*	4	1.757179	1.68 × 10^−21^	Up	24.34724562
ENSSSCT00000066335	*PDYN*	17	4.356814	9.04 × 10^−21^	Up	23.45378677
ENSSSCT00000059349	*GRK3*	14	1.179411	1.61 × 10^−16^	Up	18.56153641
ENSSSCT00000084926	Ensemble-annotated novel	4	−9.73944	2.56 × 10^−29^	Down	32.72963393
ENSSSCT00000041059	*PSMB10*	6	−1.0774	1.41 × 10^−17^	Down	19.85303546
ENSSSCT00000090288	*FANCD2*	13	−1.09977	1.58 × 10^−15^	Down	17.32346903
ENSSSCT00000005849	Ensemble-annotated novel	1	−1.87097	1.11 × 10^−14^	Down	16.30561033
ENSSSCT00000033315	*PECAM1*	12	−4.00699	1.98 × 10^−13^	Down	14.81323419

Note: Chr: chromosome.

**Table 2:** Top 10 genes calculated by three algorithms in the protein–protein interaction (PPI) network.

Gene	Up/Down	Degree	Gene	Up/Down	MCC	Gene	Up/Down	MNC
*HTR1B*	Up	9	*LOXL1*	Up	10	*LOXL1*	Up	5
*LOXL1*	Up	8	*MFAP2*	Up	10	*MFAP2*	Up	5
*MFAP2*	Up	8	*FSTL3*	Up	7	*LOXL3*	Up	2
*TRPC6*	Up	8	*S100A8*	Up	7	*LOXL4*	Up	2
*TSPAN9*	Down	8	*GJC1*	Up	7	*EFEMP2*	Up	2
*SWAP70*	Up	8	*SV2B*	Up	7	*BGN*	Up	2
*FSTL3*	Up	7	*TSPAN9*	Down	7	*ENOX1*	Up	2
*S100A8*	Up	7	*PPP2R3C*	Down	7	*ARHGAP28*	Up	2
*GJC1*	Up	7	*PRORP*	Down	7	*GRK3*	Up	2
*SV2B*	Up	7	*SWAP70*	Up	7	*FSTL3*	Up	1

**Note:** *ARHGAP28*: Rho GTPase-activating protein 28; *BGN*: biglycan; *ENOX1*: ecto-NOX disulfide-thiol exchanger 1; *EFEMP2*: *EGF*-containing fibulin extracellular matrix protein 2; *FSTL3*: follistatin-like 3; *HTR1B*: 5-hydroxytryptamine receptor 1B; *LOXL1*: lysyl oxidase-like 1; *LOXL3*: lysyl oxidase-like 3; *LOXL4*: lysyl oxidase-like 4; *GRK3*: G protein-coupled receptor kinase 3; *GJC1*: gap junction protein gamma 1; *MFAP2*: microfibril-associated protein 2; MCC: maximal clique centrality; MNC: maximum neighborhood component; *PPP2R3C:* protein phosphatase 2 regulatory subunit B gamma; *PRORP*: protein-only RNase P catalytic subunit; *SWAP70*: switching B cell complex subunit SWAP70; *S100A8*: S100 calcium-binding protein A8; *SV2B*: synaptic vesicle glycoprotein 2B; *TRPC6*: transient receptor potential cation channel subfamily C member 6; *TSPAN9*: tetraspanin 9.

**Table 3:** Top five up- and down-regulated abundant and differentially expressed microRNAs.

MicroRNA	Log_2_FoldChange	*p*-Value	Adjusted *p*-Value	Sig	−log *p*-Value
ssc-miR-146b	1.9564163	3.80 × 10^−11^	2.63 × 10^−9^	Up	10.42
ssc-miR-22-3p	2.0910663	4.50 × 10^−11^	2.63 × 10^−9^	Up	10.35
ssc-miR-24-3p	1.3624454	6.30 × 10^−10^	3.08 × 10^−8^	Up	9.20
ssc-miR-151-3p	1.9862595	1.07 × 10^−8^	3.91 × 10^−7^	Up	7.97
ssc-miR-212	1.9058319	5.82 × 10^−8^	1.82 × 10^−6^	Up	7.24
ssc-miR-2320-5p	−2.731437	6.11 × 10^−24^	1.79 × 10^−21^	Down	23.21
ssc-miR-92a	−1.737222	2.01 × 10^−19^	2.94 × 10^−17^	Down	18.69
ssc-miR-378b-3p	−2.072251	2.07 × 10^−12^	2.02 × 10^−10^	Down	11.68
ssc-miR-107	−1.476528	1.06 × 10^−8^	3.91 × 10^−7^	Down	7.97
ssc-miR-34c	−1.583839	8.51 × 10^−8^	2.08 × 10^−6^	Down	7.07

**Table 4:** miRNA–mRNA interactions identified by consensus prediction using miRanda, RNAhybrid, and RNA22. Only negatively correlated miRNA–mRNA pairs with predicted binding sites located in the 3′-untranslated region (3′UTR) and supported by all three tools, as well as the Spearman correlations, are shown.

miRNA	Up/Down	Transcripts	Gene	Up/Down	*p*-Value	r-Value	FDR	miranda_score	rnahybrid_MFE	rna22_MFE	binding_region
ssc-miR-129b	Up	ENSSSCT00000042009	*TIMP4*	Down	0.001	−0.870	0.001	−23.2	−26.9	−13.8	3’UTR
ssc-miR-132	Up	ENSSSCT00000002603	*PSEN1*	Down	0.002	−0.821	0.002	−21.3	−26.8	−12.3	3’UTR
ssc-miR-132	Up	ENSSSCT00000018375	*CCL28*	Down	0.000	−0.917	0.000	−25.9	−29.9	−12.2	3’UTR
ssc-miR-1468	Up	ENSSSCT00000002603	*PSEN1*	Down	0.001	−0.835	0.002	−23.8	−25.8	−20.2	3’UTR
ssc-miR-212	Up	ENSSSCT00000008911	*EDAR*	Down	0.000	−0.887	0.001	−24.6	−30.4	−22.7	3’UTR
ssc-miR-212	Up	ENSSSCT00000014496	*SLC35C1*	Down	0.002	−0.812	0.003	−24.7	−29.6	−18.2	3’UTR
ssc-miR-212	Up	ENSSSCT00000057862	*TIFAB*	Down	0.000	−0.894	0.001	−23.2	−30.6	−25.0	3’UTR
ssc-miR-24-1-5p	Up	ENSSSCT00000042009	*TIMP4*	Down	0.000	−0.881	0.001	−22.4	−27.6	−16.3	3’UTR
ssc-miR-24-3p	Up	ENSSSCT00000033361	*CD101*	Down	0.000	−0.890	0.001	−22.2	−29.6	−20.5	3’UTR
ssc-miR-24-3p	Up	ENSSSCT00000060396	*AVL9*	Down	0.001	−0.856	0.002	−21.5	−26.0	−18.9	3’UTR
ssc-miR-34c	Down	ENSSSCT00000042429	ENSSSCT00000042429	Up	0.001	−0.845	0.002	−21.5	−21.9	−16.2	3’UTR
ssc-miR-34c	Down	ENSSSCT00000042927	*DYNLT1*	Up	0.001	−0.843	0.002	−24.2	−28.1	−19.3	3’UTR
ssc-miR-34c	Down	ENSSSCT00000042927	*PTPRM*	Up	0.002	−0.811	0.003	−20.5	−24.6	−16.6	3’UTR
ssc-miR-34c	Down	ENSSSCT00000055918	*ALPL*	Up	0.000	−0.872	0.001	−21.5	−26.7	−19.7	3’UTR
ssc-miR-34c	Down	ENSSSCT00000062597	*ZCCHC24*	Up	0.002	−0.815	0.003	−24.9	−32.1	−23.2	3’UTR
ssc-miR-493-3p	Up	ENSSSCT00000008911	*EDAR*	Down	0.001	−0.857	0.002	−24.6	−27.8	−21.2	3’UTR
ssc-miR-493-3p	Up	ENSSSCT00000046745	*SNX4*	Down	0.000	−0.903	0.001	−21.8	−26.4	−13.2	3’UTR

Note: **MFE:** minimum free energy.

## Data Availability

Raw sequence data were deposited in GenBank of the National Center for Biotechnology Information (NCBI) (accession number PRJNA916351).

## Supplementary Materials

The following supporting information can be downloaded at: https://www.mdpi.com/article/10.3390/biology15141123/s1, Table S1. Overview and quality control for the RNA sequencing data; Table S2. Summary of reads mapped to reference genome based on mRNA sequencing; Table S3. Overview and quality control for the miRNA sequencing data; Table S4. Summary of reads mapped to the reference genome based on miRNA sequencing; Table S5. Top KEGG pathway-based sets of differentially expressed genes (DEGs) between CON and DSS groups; Figure S1. Venn plot of the up-regulated genes (A) and down-regulated genes (B) enriched in the top four or top three KEGG pathways; Figure S2. Venn diagram showing overlaps of miRNA-mRNA interactions predicted by miRanda, RNAhybrid, and RNA22 after filtering for inverse expression correlation and 3′UTR binding sites; Supplemental File S1. The information about the new transcripts is named MSTRG; Supplemental File S2. Various transcripts with different expression levels for the six genes; Supplemental File S3. The information on all the transcripts with expression levels in the samples; Supplemental File S4. Gene ontology (GO) enrichment analysis of the differentially expressed transcripts; Supplemental File S5. Kyoto Encyclopedia of Genes and Genomes (KEGG) enrichment analysis of the differentially expressed transcripts; Supplemental File S6. Identified miRNA expression information in all the samples; Supplemental File S7. Positively and negatively interacted transcript-miRNA pairs.

## Abbreviations

*ASIC1*	acid-sensing ion channel subunit 1
*ARHGEF28*	Rho guanine nucleotide exchange factor 28 gene
*AKT3*	AKT serine/threonine kinase 3 gene
BP	biological process
*CCL28*	C-C motif chemokine ligand 28 gene
*CCR10*	C-C motif chemokine receptor 10 gene
*CCL5*	C-C motif chemokine ligand 5 gene
CC	cellular component
*CXCL14*	C-X-C motif chemokine ligand
*COL6A1*	collagen type VI alpha 1 chain gene
*COL18A1*	collagen type XVIII alpha 1 chain gene
CON	control group
CDS	coding sequences
3’UTR	3’ untranslated region
5’UTR	5’ untranslated region
DSS	dextran sodium sulfate
DE	differentially expressed
ECM	extracellular matrix
FPKM	fragments per kilobase of transcript per million mapped reads
FDR	false discovery rate
*FSTL3*	follistatin-like 3
GO	gene ontology
IBD	inflammatory bowel disease
KEGG	Kyoto Encyclopedia of Genes and Genomes
*LOXL1*	lysyl oxidase-like 1
miRNAs	microRNAs
MEF	minimum free energy
MFAP2	microfibril-associated protein 2
MMPs	matrix metalloproteinases
MF	molecular functions
NGS	next-generation sequencing
*PIK3CB*	phosphatidylinositol-4,5-bisphosphate 3-kinase catalytic subunit beta gene
*PIK3R1*	phosphoinositide-3-kinase regulatory subunit 1 gene;
*PSMB10*	proteasome 20S subunit beta 10
*PCDHGA4*	protocadherin gamma subfamily A 4
PCA	principal component analysis
PPI	protein–protein interaction
*RAF1*	Raf-1 proto-oncogene, serine/threonine kinase gene
*SNX4*	sorting nexin 4 gene
*STAT1*	signal transducer and activator of transcription 1 gene
*TNFSF13*	TNF superfamily member 13 gene
*TNFRSF17*	TNF superfamily member 17 gene
UC	ulcerative colitis
