# Supplementary material for: Analysis of Colon Transcriptomes in a Porcine Model of Dextran Sodium Sulfate (DSS)-Induced Ulcerative Colitis
Source: Biology (Basel). 2026 Jul 10;15(14):1123. doi: 10.3390/biology15141123 (PMC13405138; doi:10.3390/biology15141123)
Supplement: Supplementary file 1 [file biology-15-01123-s001.zip › Figures.pdf]

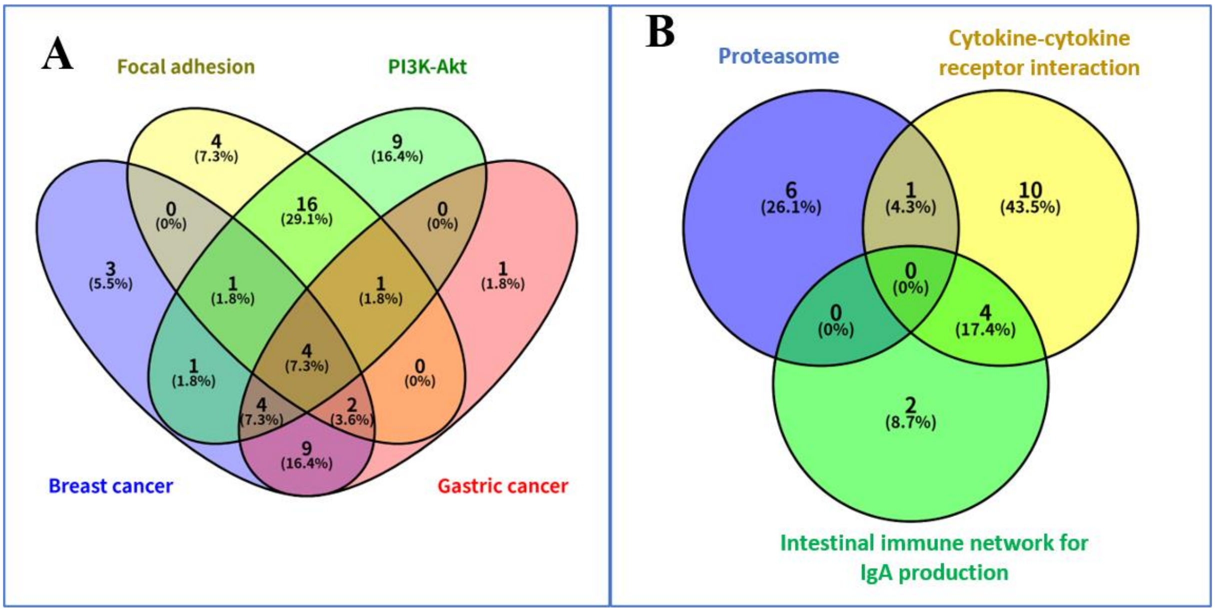


**Figure S1**. Venn plot of the up-regulated genes (A) and down-regulated genes (B) enriched in the top four or top three KEGG pathways.

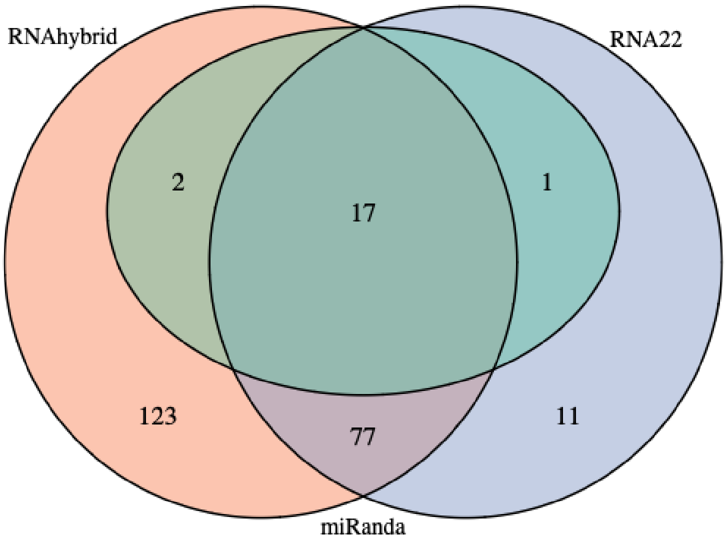


**Figure S2.** Venn diagram showing overlap of miRNA-mRNA interactions predicted by miRanda, RNAhybrid, and RNA22 after filtering for inverse expression correlation and 3′UTR binding sites.
