# Supplementary material for: Analysis of Colon Transcriptomes in a Porcine Model of Dextran Sodium Sulfate (DSS)-Induced Ulcerative Colitis
Source: Biology (Basel). 2026 Jul 10;15(14):1123. doi: 10.3390/biology15141123 (PMC13405138; doi:10.3390/biology15141123)
Supplement: Supplementary file 1 [file biology-15-01123-s001.zip › TablesS_DSS_CON.pdf]

Table S1. Overview and quality control for the RNA sequencing data

ID	Raw data	Q20	Q30	GC%	LQ [a]	TMN [b]	TST[c]	Clean data
CON_27	76,929,870	97.21%	91.63%	50.71%	121,548 (0.16%)	1,766 (0.002%)	992,74	75,813,808 (98.55%)
CON_31	104,232,126	95.74%	89.57%	50.57%	188,594 (0.18%)	0 (0.000%)	1,497,918	102,545,614 (98.38%)
CON_37	76,699,454	96.03%	90.10%	49.64%	100,474 (0.13%)	0 (0.000%)	937,654	75,671,326 (98.66%)
CON_38	74,395,700	97.38%	93.04%	51.70%	86,198 (0.12%)	2,482 (0.003%)	686,900	73,620,120 (98.96%)
CON_45	70,431,088	97.29%	92.85%	51.71%	84,652 (0.12%)	2,462 (0.003%)	687,150	69,656,824 (98.9%)
CON_46	70,574,060	96.03%	90.03%	50.36%	78,490 (0.11%)	3,672 (0.005%)	753,182	6,973,816 (96.6%)
DSS_29	77,942,940	96.11%	90.07%	49.67%	113,484 (0.15%)	1,650 (0.002%)	985,520	76,842,286 (99.59%)
DSS_32	92,790,650	95.85%	89.80%	51.66%	127,196 (0.14%)	0 (0.000%)	1,095,546	91,567,908 (98.68%)
DSS_39	71,431,272	97.66%	93.63%	50.50%	64,658 (0.09%)	2356 (0.003%)	566,298	70,797,960 (90.11%)
DSS_40	86,136,734	95.88%	89.78%	49.27%	110,244 (0.13%)	0 (0.00%)	1,057,190	84969300 (98.6%)
DSS_47	70396736	97.33%	92.83%	50.23%	74,722 (0.11%)	2,330 (0.003%)	630,382	69,689,302 (93.7%)

Note: [a]: means the reads with low quality; [b]: means the reads with too many N; [c]: means the read too short

Table S2. Summary of reads mapped to reference genome based on mRNA sequencing.

Mapping	CON_27	CON_31	CON_37	CON_38	CON_45	CON_46	DSS_29	DSS_32	DSS_39	DSS_40	DSS_47
NPH	11,769,128(13.44%)	9,421,687(8.41%)	8,011,596(9.57%)	15,430,20(17.33%)	12,169,284(14.87%)	12,610,012(15.31%)	8,391,151(9.84%)	12,254,561(11.80%)	6,780,566(8.74%)	7883694(8.49%)	8,341,468(10.69%)
UR	3,025,466(3.45%)	4,913,263(4.39%)	4,481,925(5.36%)	2,348,697(2.64%)	3,776,700(4.62%)	3,682,405(4.47%)	3,080,862(3.61%)	5,089,959(4.90%)	7,688,920(9.91%)	4,621,187(4.98%)	5,251,193(6.73%)
MQ<MC	7,247,327(8.27%)	5,901,440(5.27%)	4,932,530(5.89%)	9,017,366(10.13%)	7,566,529(9.25%)	7,410,741(9.00%)	5,474,510(6.42%)	7,756,183(7.47%)	4,658,551(6.00%)	5,177,254(5.58%)	5,106,637(6.54%)
MQ>=MC	65,541,015(74.83%)	91,730,911(81.93%)	66,256,871(79.18%)	62,254,057(69.91%)	58,313,595(71.27%)	58,645,570(71.22%)	68,286,914(80.12%)	78,721,766(75.82%)	58,450,489(75.34%)	75,170,859(80.96%)	59,331,472(76.04%)
Read-1	32,972,031(37.65%)	46,346,858(41.39%)	33,383,430(39.89%)	31,191,755(35.03%)	29,218,415(35.71%)	29,511,698(35.84%)	34,313,903(40.26%)	39,656,247(38.20%)	29,254,072(37.71%)	37,888,009(40.80%)	29,719,634(38.09%)
Read-2	32,568,984(37.19%)	4,5384,053(40.53%)	32,873,441(39.28%)	31,062,302(34.88%)	29,095,180(35.56%)	29,133,872(35.38%)	33,973,011(39.86%)	39,065,519(37.63%)	29,196,417(37.63%)	37,282,850(40.15%)	29,611,838(37.95%)
RP(+)	32,740,944(37.38%)	45,797,188(40.90%)	33,095,760(39.55%)	31,110,752(34.94%)	29,129,450(35.60%)	29,291,761(35.57%)	34,112,130(40.02%)	39,293,599(37.85%)	29,217,601(37.66%)	37,537,575(40.43%)	29,648,737(38.00%)
RP(-)	32,800,071(37.45%)	45,933,723(41.02%)	33,161,111(39.63%)	31,143,305(34.97%)	29,184,145(35.67%)	29,353,809(35.65%)	34,174,784(40.10%)	39,428,167(37.98%)	29,232,888(37.68%)	37,633,284(40.53%)	29,682,735(38.04%)
NPR	42,448,262(48.47	56,907,226(50.82	44,568,170(53.26	41,562,285(46.67	41,700,203(50.96	39,885,922(48.44	49,183,081(57.70	55,047,199(53.02	39,021,226(50.30	53,862,204(58.01	39,985,258(51.24

	%)	%)	%)	%)	%)	%)	%)	%)	%)	%)	%)
SR	23,092,753(26.37%)	34,823,685(31.10%)	21,688,701(23.24%)	20,691,772(23.24%)	16,613,392(20.30%)	18,759,648(22.78%)	19,103,833(22.41%)	23,674,567(22.80%)	19,429,263(25.04%)	21,308,655(22.95%)	19,346,214(24.79%)
RMPP	63,795,258(72.84%)	88,996,176(79.48%)	64,185,450(76.70%)	60,764,018(68.24%)	56,868,034(69.50%)	56,684,316(68.83%)	66,425,970(77.93%)	76,364,816(73.55%)	57,221,808(73.76%)	72,751,682(78.53%)	57,884,366(74.18%)
Total reads	87,582,936(100%)	111,967,301(100%)	83,682,922(100%)	89,050,325(100%)	81,826,108(100%)	82,348,728(100%)	85,233,437(100%)	103,822,469(100%)	77,578,526(100%)	92,852,994(100%)	78,030,770(100%)

Note: NPH: Non primary hits; UR: Unmapped reads; MQ<MC: mapq < mapq_cut (non-unique); MQ>=MC: mapq >= mapq_cut (unique); RP (+): Reads map to '+'; RP (-): Reads map to '-'; NPR: Non-splice reads; SR: Splice reads; RMPP: Reads mapped in proper pairs

Table S3. Overview and quality control for the microRNA sequencing data

Sample ID	Raw data	LQ	Q20	Q30	GC %	5'primer C	3'primer C	Length<16	Length >35	Clean data
CON_27	20,462,957	0.60%	97.36%	95.05%	49.00%	87,619 (0.43%)	334,868 (1.63%)	366,142(1.8%)	278,760(1.4%)	19,818,055 (96.8%)
CON_31	20,750,439	0.50%	97.59%	95.40%	49.24%	64,432 (0.35%)	231,437 (1.11%)	403,419 (1.9%)	275,968 (1.3%)	20,071,052 (96.7%)
CON_37	21,464,522	0.39%	97.64%	95.33%	49.41%	76,147 (0.35%)	185,315 (0.89%)	1,032,167 (4.8%)	204,103 (1.0%)	20,228,252 (94.2%)
CON_38	21,032,273	0.40%	97.75%	95.63%	49.16%	60,729 (0.29%)	201,699 (0.96%)	804,421(3.8%)	142,729 (0.7%)	20,085,123(95.5%)
CON_45	20,906,793	0.42%	97.62%	95.38%	49.86%	81,564 (0.39%)	207,498 (0.99%)	651,142 (3.1%)	214,556 (1.0%)	20,041,095 (95.9%)
CON_46	22,252,493	0.36%	97.78%	95.69%	49.09%	68,521 (0.31%)	177,058 (0.79%)	267,910 (1.2%)	134,179 (0.6%)	21,944,119 (96.6%)
DSS_29	20,644,594	0.67%	97.15%	94.69%	49.28%	81,100 (0.39%)	280,964 (1.36%)	2,771,416 (8.6%)	254,737 (1.2%)	18,618,441 (90.2%)
DSS_32	20727569	0.36 %	97.63%	95.33%	50.03%	31,502 (0.15%)	670,403 (3.23%)	1524,416 (7.3%)	184,737 (8.9%)	19,943,350 (96.22%)
DSS_39	21,021,479	0.27%	97.64%	95.23%	49.36%	139,353 (0.66%)	154,428 (0.73%)	1,117,098 (5.3%)	275,960 (1.3%)	19,628,421 (93.4%)
DSS_40	20,163,457	0.39%	97.60%	95.23%	48.89%	108,070 (0.54%)	169,459 (0.84%)	1,549,337 (7.7%)	132,318 (0.7%)	18,481,802 (91.7%)
DSS_47	22,918,393	1.87%	95.73%	92.74%	49.61%	164,817 (0.38%)	1,284,119 (2.99%)	1,771,010 (4.1%)	93,542 (2.2%)	21,474,534 (93.7%)

Note: C: Contaminants

Table S4. Summary of reads mapped to the reference genome based on microRNA sequencing

Sample	Total sRNA	Mapped sRNA	'+' Mapped sRNA	'-'Mapped sRNA
CON_27	19,818,055 (100%)	11,470,676 (57.88%)	10,135,937 (51.14%)	1,334,739 (6.73%)
CON_31	20,071,052 (100%)	11,722,533 (58.41%)	10,713,722 (53.38%)	1,008,811(5.03%)
CON_37	20,228,252 (100%)	11,573,798 (57.22%)	10,614,076 (52.47%)	959,722 (4.74%)
CON_38	20,085,123 (100%)	12,373,335 (61.60%)	11,499,563 (57.25%)	873,772 (4.35%)
CON_45	20,041,095 (100%)	10,604,417 (52.92%)	8,506,245 (42.44%)	1,098,172 (5.48%)
CON_46	21,944,119 (100%)	11,574,369 (52.74%)	10,584,793 (48.24%)	989,576 (4.51%)
DSS_29	18,618,441 (100%)	12,255,074 (65.82%)	10,865,498 (58.36%)	1,389,576 (7.46%)
DSS_32	20977474 (100%)	15836221 (75.49%)	14277706 (68.06%)	1558515 (7.43%)
DSS_39	19,628,421 (100%)	14,533,253 (74.04%)	13,256,758 (67.54%)	1,276,495 (6.50%)
DSS_40	18,481,802 (100%)	14,441,452 (78.14%)	13,030,751 (70.51%)	1,410,701 (7.63%)
DSS_47	40,211,959 (100%)	30,102,412 (74.86%)	27,203,594 (67.65%)	2,898,818 (7.21%)

Table S5. Top KEGG pathway-based sets of differentially expressed genes (DEGs) between CON and DSS group

Description	ID	Count	*P*-adjust	geneID	Up_down
Breast cancer	ssc05224	24	1.37E-08	*IGF1/NOTCH4/HEY2/FZD7/FGF10/FGF1/RAF1/FGFR1/PIK3R1/SHC1/WNT5A/LRP6/FGF2/AKT3/WNT2/GADD45A/WNT9B/SHC4/LEF1/FGF7/PIK3CB/WNT6/DLL1/GADD45B*	Up
Focal adhesion	ssc04510	28	1.37E-08	*PARVB/IGF1/THBS1/LAMC3/RAPGEF1/SPP1/COL4A2/COMP/LAMC1/ITGAV/RAF1/PIK3R1/SHC1/KDR/LAMB2/AKT3/TNN/COL2A1/LAMA1/SHC4/COL6A3/PIK3CB/HGF/LAMB1/ACTG1/COL4A1/THBS4/ROCK2*	Up
PI3K-Akt signaling pathway	ssc04151	36	1.63E-07	*IGF1/THBS1/LAMC3/AREG/SPP1/COL4A2/CREB3L3/COMP/LAMC1/ITGAV/FGF10/CSF3/IL6/FGF1/RAF1/FGFR1/PIK3R1/KDR/FGF2/TSC2/LAMB2/AKT3/TNN/COL2A1/LAMA1/FGF7/COL6A3/PIK3CB/HGF/LAMB1/MAGI2/ANGPT2/CSF3R/COL4A1/THBS4/OSMR*	Up
ECM-receptor interaction	ssc04512	16	2.29E-06	*THBS1/LAMC3/SPP1/COL4A2/COMP/LAMC1/ITGAV/LAMB2/TNN/COL2A1/LAMA1/SV2B/COL6A3/LAMB1/COL4A1/THBS4*	Up
amoebiasis	ssc05146	16	1.02E-05	*ARG1/LAMC3/ITGAM/CXCL8/COL4A2/CD14/LAMC1/IL6/PIK3R1/TGFB3/LAMB2/LAMA1/PIK3CB/LAMB1/IL1R1/COL4A1*	Up
Proteasome	ssc03050	7	0.003705552	*PSMB8/PSMB9/PSMB10/IFNG/PSMA6/PSMA4/PSME1*	Down
Cytokine-cytokine receptor interaction	ssc04060	15	0.012486133	*XCL1/EDAR/CXCR6/MSTN/CCL28/FASLG/CXCR3/CCL5/CCL8/TNFSF13/IFNG/CXCL9/ACVR1C/CCR10/TNFRSF17*	Down
Intestinal immune network for IgA production	ssc04672	6	0.020131167	*SLA-DMB/PIGR/CCL28/TNFSF13/CCR10/TNFRSF17*	Down
